# The serum levels of circulating matrix metalloproteinase MMP-9, MMP-2/TIMP-2 complex and TIMP-1 do not change significantly during normal pregnancy: a pilot study

**DOI:** 10.1186/s13104-021-05442-w

**Published:** 2021-01-22

**Authors:** Ritva Nissi, Markku Santala, Anne Talvensaari-Mattila

**Affiliations:** Department of Obstetrics and Gynecology, Oulu University Hospital, PO Box 5000, 90014 Oulu, Finland

**Keywords:** Matrix metalloproteinases (MMPs), Tissue inhibitors of matrix metalloproteinases (TIMPs), Pregnancy

## Abstract

**Objective:**

Matrix metalloproteinases (MMPs) are important regulators of vascular and uterine remodeling. They exhibit proteolytic activity implicating the efficiency of trophoblast invasion to the uterine wall involving marked hemodynamic and uterine changes. In this pilot study sera of 13 women with normal pregnancy was analyzed to evaluate the usage of MMPs as diagnostic tool. The concentrations of circulating MMP-9, MMP-2/TIMP-2 complex and TIMP-1 in different time points during normal pregnancy has not been studied. The serum levels of MMP-9, TIMP-1, TIMP-2 and MMP-2/TIMP-2 complex were determined by enzyme-linked immunosorbent assay (ELISA). Using the same method, we have shown that serum MMPs are elevated in spontaneous early pregnancy failure as compared to normal pregnancy.

**Results:**

The serum levels of MMP-9 and TIMP-1 were stable throughout pregnancy. The level of MMP-2/TIMP-2 complex was slightly increased after week 15 without statistical significance. For our best knowledge, this is a first study of the serum levels of MMP-9, MMP-2/TIMP-2 and TIMP-1 on different time points during normal pregnancy. Further measurements with the correlation to the outcome of the pregnancy are needed.

## Introduction

Matrix Metalloproteinases (MMPs) consists of a large family of at least 28 proteolytic enzymes. MMPs are structurally related, zinc-dependent endopeptidases, which hydrolyze extracellular matrix components collagen being a main substrate. MMPs involves processes like embryogenesis and implantation, wound healing, inflammatory states, tumor metastasis, angiogenesis and various other pathological conditions. MMPs can be inactivated through tissue inhibitors of metalloproteinases (TIMPs) [1].

The gelatinases MMP-2 and MMP-9 are especially involved in successful cytotrophoblast invasion in early pregnancy as they are considered key enzymes of degradation of basement membrane. Transcription and secretion are thought to increase in preparation for labor, resulting in cervical ripening, and dilation and subsequent rupture of the fetal membranes [2].

The amount and activity of MMP-2 and MMP-9 are increased in the aorta of normal pregnant rats, supporting a role of MMPs in pregnancy-associated vascular remodeling [3]. MMP-9 knockout mice shows a phenotype mimicking preeclampsia [4]. Measurements of the plasma levels of MMPs have not been consistent in preeclampsia: some studies show an increase in serum levels of MMP-2 and MMP-9, whereas some studies show a decreased MMP-9 level [1]. Serum levels of MMP-2, MMP-9 and their inhibitors do not differ between pregnant woman with glucose intolerance as compared to healthy controls [5]. Serum imbalances between matrix metalloproteinases and their inhibitors have been detected in preterm labor [6]. The changes in serum levels of MMP-9, MMP-2 and their respective tissue inhibitors TIMP-1 and TIMP-2 in different time points has not been studied in normal pregnancy and measurements of the plasma levels of MMPs have not been consistent between studies in complicated pregnancies in all studies. In this pilot study we analyzed sera of 13 women with normal pregnancy to evaluate the usage of MMPs as diagnostic tool in complicated pregnancies.

## Main text

### Methods

The study was conducted in Oulu University Hospital in the department of Obstetrics and Gynecology. 13 patients were enrolled in this study. The patients who participate the study come to their first visit to maternity clinic on pregnancy week 10. They are also followed in weeks 15–16, 26–28 and 36–37. Every visit patient attended, blood samples were taken to assess matrix metalloproteinases. Venous blood samples were collected after ultrasound examination. Sera were obtained by centrifugation without using any artificial coagulation activator and stored frozen at − 20 °C until analysis for this study.

**Fig. 1:**
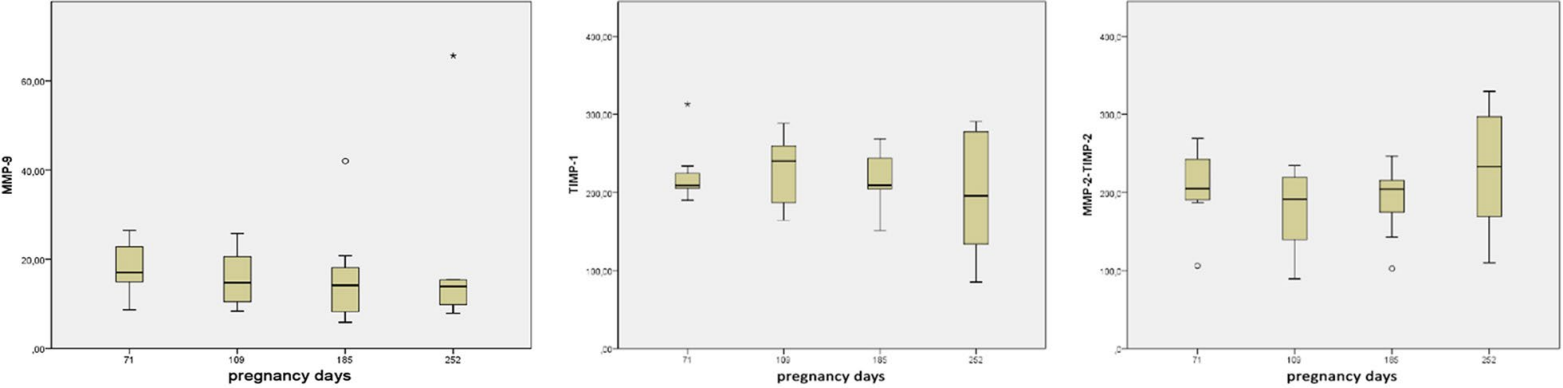
Box-plot medians and ranges of MMP-9, TIMP-1 and MMP-2-TIMP-2 complex in maternal serum of different stages of pregnancy

The concentrations of MMP-9, TIMP-1, TIMP-2 and MMP-2/TIMP-2 complex in the serum of the study patients were determined by enzyme-linked immunosorbent assay (ELISA). ELISA assays were performed on 8-well EIA/RIA microtiter plates (Corning Inc., Corning, NY, USA) using standard protocols [7]. Standard samples were included in every plate and the standard curves were required to be similar in each lot. All measurements were performed in duplicate. The wells were coated overnight at 4 °C with a specific monoclonal antibody provided by SBA Sciences, Oulu, Finland (code DB-102 for TIMP-1, code T2-101 for TIMP-2 and MMP-2/TIMP-2, code Ge-213 for MMP-9). Following coating, diluted serum samples and standards for TIMP-1, TIMP-2 and MMP-2/TIMP-2 complex was incubated for 60 min, or overnight in the case of MMP-9. Non-specific binding was blocked with phosphate-buffered saline containing 1% bovine serum album (BSA-PBS). The wells were washed thoroughly before each stage of the procedure, in the first phase with PBS and in the later stages with PBST (0.05% Tween 20 in PBS). The bound proteins were detected with polyclonal antibodies against each of the analyses (anti-TIMP-1, code DB-205 for TIMP-2, code DB-202 for MMP-2/TIMP-2 complex, code DB-209 for MMP-9) (SBA Sciences, Oulu, Finland). A peroxidase conjugated anti-chicken antibody (Chemicon International, CA, USA) was used to detect the bound polyclonal antibody, and an OPD solution (*o*-phenylenediamine dihydrochloride, P-1526; Sigma, Steinheim, Germany) was used to visualize the peroxidase conjugate. The reaction was stopped with 1.8 M H_2_SO_4_. Color formation was measured at 492 nm with a microplate reader (Anthos Reader 2001; Anthos Labtec Instruments, Walls, Austria) using the Windows-based control and evaluation software for Rosys Anthos microplate readers (Anthos Labtec Instruments). The sensitivity of the assays was 2 ng/mL for MMP-9, 1 ng/mL for TIMP-1, 2 ng/mL for TIMP-2 and 2 ng/mL for MMP-2/TIMP-2 complex. The laboratory data was analyzed statistically using Matlab 7.0.4.365 for Windows using Kruskal–Wallis test (Fig. 1).

### Results

Patients median age was 31 (23–40). As shown on Table 1, median MMP-9 levels were 19 ng/ml on week 10, 15 ng/ml on week 15–16, 14 ng/ml on week 26–28 as well as week 36–37. For MMP-9, no marked changes were observed. For TIMP-1 median values were 212 ng/ml on week 10, 240 ng/ml on week 15–16, 209 ng/ml on week 26–28 and 196 ng/ml on week 36–37. For MMP-2/ TIMP-2 complex, the values were 199 ng/ml on week 10, 191 ng/ml on week 15–16, 204 ng/ml on week 26–28 and 233 ng/ml on week 36–37. An increased TIMP-2 level on women with a history of recurrent pregnancy loss has been observed [6].

**Table 1:** Comparison of TIMP-1, MMP-2/TIMP2 and MMP-9 in maternal serum

Protein	n	Pregnancy week 10 (n = 18)	Pregnancy weeks 15–16 (n = 31)	Pregnancy weeks 26–28 (n = 27)	Pregnancy weeks 36–37 (n = 24)
TIMP-1	33	212 (204–313)	240 (164–288)	208 (151–269)	196 (85–291)
MMP-2-TIMP-2	33	199 (106–269)	191 (89–235)	204 (102–246)	233 (110–329)
MMP-9	34	19 (14–26)	15 (8–26)	14 (6–42)	14 (8–64)

Results are expressed as median (range) (ng/ml)

There are only few studies on MMPs or TIMPs in maternal serum. Lakowska [8] reported that decreased MMP-9 levels may be involved on pathological processes during pregnancy such as fetal growth restriction (FGR) and preeclampsia, but the results are not matched on pregnancy week.

### Conclusion

Our results show no marked changes when measuring MMP-9, TIMP-1 and MMP-2/TIMP-2 serum levels on different time points during normal pregnancy. The levels of MMP-2/TIMP-2 complex increased from pregnancy weeks 15–16 forward but the difference was not statistically significant. On the other hand, during pregnancy the blood volume increases: that may mix the results. Our observations suggest that cytokine changes during pregnancy are not reflected in maternal circulation and alterations in cytokine profiles are strictly compartmentalized and independently regulated.

## Limitations

There are only few studies how serum levels of matrix metalloproteinases and their tissue inhibitors change during normal pregnancy. The contribution of reproductive tract tissue to serum levels of MMPs and TIMPs during pregnancy is unknown. The main limitations in this study is our small sample size and lack of comparison group. Future studies are needed.

## Data Availability

All data generated in the present study is available from the authors on reasonable request.

## Abbreviations

MMPs: Matrix metalloproteinases
MMP-9: Matrix metalloproteinase 9
MMP-2: Matrix metalloproteinase 2
TIMP-1: Tissue inhibitor of matrix metalloproteinase 1
TIMP-2: Tissue inhibitor of matrix metalloproteinase 2
FGR: Fetal growth restriction
